# Supplementary material for: Fermi level position, Coulomb gap, and Dresselhaus splitting in (Ga,Mn)As
Source: Sci Rep. 2016 Jun 6;6:27266. doi: 10.1038/srep27266 (PMC4893692; doi:10.1038/srep27266)
Supplement: Supplementary Information [file srep27266-s1.pdf]

SUPPLEMENTARY INFORMATION

Fermi level position, Coulomb gap, and Dresselhaus splitting in (Ga,Mn)As

S. Souma[1], L. Chen[1], R. Oszwałdowski[2], T. Sato[3], F. Matsukura[1,4,5], T. Dietl[1,6,7], H. Ohno[1,4,5], & T. Takahashi[1,3].

[1]*WPI Research Center, Advanced Institute for Materials Research, Tohoku University, 2-1-1 Katahira, Aoba-ku, Sendai 980-8577, Japan*

[2]*Department of Physics, South Dakota School of Mines and Technology, Rapid City, SD 57701, USA*

[3]*Department of Physics, 6-3 Aramaki Aza-Aoba, Aoba-ku, Tohoku University, Sendai 980-8578, Japan*

[4]*Center for Spintronics Integrated Systems, Tohoku University, 2-1-1 Katahira, Aoba-ku, Sendai 980-8577, Japan*

[5]*Laboratory for Nanoelectronics and Spintronics, Research Institute of Electrical Communication, Tohoku University, 2-1-1 Katahira, Aoba-ku, Sendai 980-8577, Japan*

[6]*Institute of Physics, Polish Academy of Sciences, aleja Lotników 32/46, PL-02-668 Warszawa, Poland*

[7]*Institute of Theoretical Physics, Faculty of Physics, University of Warsaw, ulica Pasteura 5, PL-02-093 Warszawa, Poland.*

1. Band assignment of GaAs and photoelectron matrix-element effect

We observed a strong selection rule of photoelectron intensity[40] (*i.e.* photoemission matrix-element effect) in the ARPES data of GaAs and (Ga,Mn)As. As displayed in Fig. S1**a**, when the polarization vector of monochromatized He-Iα resonance line points perpendicular to the measured $\overline{\Gamma X}$ cut (see inset), both the light hole (LH) and heavy hole (HH) bands are well resolved, particularly in high binding-energy region, while the intensity of the split-off (SO) band appears to be very weak. On the other hand, when the polarization vector is aligned parallel to the $\overline{\Gamma X}$ cut as shown in Fig. S1**b**, the SO band appear to be more visible, while the LH/HH bands become dimmer. This characteristic polarization dependence of ARPES intensity is consistent with the previous report of soft-x-ray ARPES on (Ga,Mn)As[23].

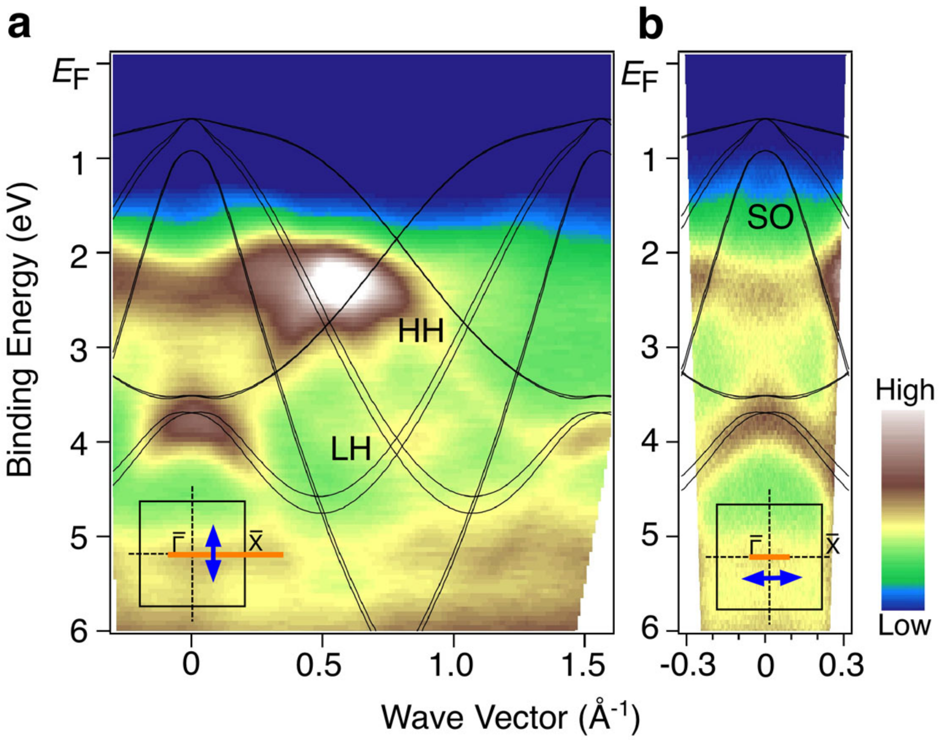

Figure S1 l Light-polarization dependence of ARPES intensity of GaAs. a, ARPES intensity of GaAs (n-type; Si-doped) measured along the ΓX̄ cut by setting the polarization vector of incident light perpendicular to the measured cut (see arrow in inset). **b**, ARPES intensity of GaAs measured with the polarization vector parallel to the measured cut (inset). Calculated band structure of GaAs within the tight-binding approximation (same as Fig. 1e) is overlaid by solid curves in **a** and **b**.
